# Editorial: GPCRs: signal transduction

**DOI:** 10.3389/fmolb.2024.1403161

**Published:** 2024-04-10

**Authors:** Nicole Perry-Hauser, Stuart Maudsley, Yamina A. Berchiche, Lauren M. Slosky

**Affiliations:** ^1^ Columbia University Vagelos College of Physicians and Surgeons, New York City, NY, United States; ^2^ New York State Psychiatric Institute, New York City, NY, United States; ^3^ Receptor Biology Lab, Department of Biomedical Sciences, University of Antwerp, Antwerp, Belgium; ^4^ Dr. GPCR, Malden, MA, United States; ^5^ Department of Pharmacology, University of Minnesota Twin Cities, Minneapolis, MN, United States

**Keywords:** GPCR (G protein coupled receptor), GRK (G protein-coupled receptor kinase), RGS (regulator of G protein-signaling proteins), β-arrestin, signaling, signaling pathways

G protein-coupled receptors (GPCRs) are the largest family of transmembrane receptors in the human genome and the largest class of targets of FDA-approved therapeutics. GPCRs respond to a panoply of stimuli, including photons, small molecules, and peptides, and relay this information across the cell membrane. GPCRs translate these extracellular signals into coordinated intracellular responses, resulting in effects at the cellular, tissue, and whole organism level. The versatility of GPCR signaling in human physiology has led to their widespread exploitation for therapeutic purposes. GPCR-targeted agents represent more than a third of current pharmacotherapies. Despite decades of research into GPCR structure and function and GPCR-regulated physiological and behavioral effects, we continue to learn more about how these receptor systems function and can be targeted to produce beneficial effects. In this Research Topic, “*GPCRs: Signal Transduction*,” we present a compilation of reports that highlight new paradigms in GPCR signaling and showcase the breadth of levels at which this signaling can be studied.

The manuscripts in this collection explore GPCR signaling (Birch et al., Matt et al., Qiu et al., Pogorelov et al.), adaptor proteins that shape this signaling (Ye et al.), and regulatory proteins that integrate G protein and non-G protein-linked signals (Tian et al. and Yang et al.). These reports set the stage for an understanding of GPCR function in both health and disease. For example, Birch et al. investigated how activated protein C (APC) interacts with the GPCR protease-activated receptor-1 (PAR1) to induce cytoprotective responses in endothelial cells. PAR1 β-arrestin2 activation is a promising target for the treatment of vascular diseases, but the mechanism by which this complex elicits its cytoprotective responses is unknown. Birch et al. report that APC alters the phosphorylation of TNF-α signal pathway components, reducing TNF-α-induced expression of the inflammation-related marker VCAM-1. Unexpectedly, Birch et al. also find that different APC/PAR1 cytoprotective responses are mediated by discrete β-arrestin-2-mediated signaling pathways, driven by the presence of co-receptors and GPCR kinases (GRKs).

Behavioral consequences of functional selectivity or biased GPCR signaling are highlighted by Pogorelov et al. Pogorelov et al. examined the behavioral effect of serotonin 2A receptor (5-HT2AR) activation by two ligands: lisuride (marketed as Dopergin for Parkinson’s disease) and LSD (lysergic acid diethylamide). Although lisuride and LSD are both high-affinity 5-HT2AR partial agonists and share structural similarities, only LSD induces hallucinogenic effects in healthy subjects at routine doses. At the 5-HT2AR, lisuride is G protein-biased, while LSD is β-arrestin2-biased. Pogorelov et al. report that, in mouse models, lisuride exerted anti-depressant drug-like responses without hallucinogenic-like activities, including in β-arrestin1 and β-arrestin2 KO mice. These data suggest that β-arrestin1 and β-arrestin2 play minor roles in many of lisuride’s behavioral effects and that lisuride’s beneficial actions may be a result of G protein-biased 5-HT2AR signaling.

The role of GPCR regulatory proteins in cancer was the subject of several reports in this Research Topic. Ye et al. examined the expression of β-arrestin1 (ARRB1), a key GPCR adaptor, in 33 cancer types. Ye et al. found that ARRB1 expression was lower in almost 50% of tumors assess, as compared to non-cancerous tissues. ARRB1 expression correlated with cancer prognosis, tumor-immune infiltration, and response to immunotherpay for some cancer types. The authors conclude that ARRB1 levels may serve as a prognostic biomarker in cancers such as kidney renal clear cell carcinoma and lung adenocarcinoma and be used to predict response to immune-based cancer therapeutics. In another study by Yang et al. systematically investigated the impact of GPCR-regulating RGS proteins in gastric cancer. They found that RGS22, RGS3, RGS4, and RGS5 commonly exhibit copy number amplifications in gastric cancer. Elevated expression of RGS1, RGS2, and RGS3 was associated with poor disease prognosis, with RGS5 linked to abnormal vascular formation. Specifically, they highlighted the role of RGS4 in controlling fibroblast infiltration and epithelial-mesenchymal transition. The pathophysiological relevance of RGS16 in cancer, inflammation, and metabolic disorders is reviewed by Tian et al. The authors synthesized work on the contribution of RGS16 to cancer-related immune, inflammatory, and metabolic processes. RGS16-regulated signaling pathways in these disease states include MAPKs (mitogen-activated protein kinases), PI3Ks/PKB (phosphoinositide 3-kinase/protein kinase B), ROHA (Ras homolog family member A), and SDF-1/CXCR4 (stromal cell-derived factor 1/C-X-C motif chemokine receptor four).

Moving from assessments of GPCR adapters and regulatory proteins back to the receptors themselves, Matt et al. investigated the signaling diversity of β-adrenergic receptors (β-ARs) expressed in the central nervous system. Matt et al. used ligand-induced Emax (maximal response level) and EC50 (half maximal effective concentration) in conjunction with reference agonist values to create system-independent “fingerprints” for β-AR subtypes. These fingerprints were then used to assess receptor subtype expression across human brain cell systems. β2-AR functional expression was identified across several human brain cell types. The final report in this Research Topic highlights the power of GPCR screening for therapeutic discovery. With the goal of identifying new targets for the improvement of cardiovascular health. Qiu et al. undertook a screen to identify GPCRs controlling expression of a zinc-finger transcription factor that promotes endothelium health: KFL2 (Krüppel-like Factor 2). This screen identified several novel anti-atherosclerotic GPCR loci, including GPR116, SSTR3, GPR101, and LGR4. Qiu et al. go on to describe their drug discovery efforts surrounding LGR4, highlighting its potential as a therapeutic target for improving endothelial function in cardiovascular disease.

To conclude, research on GPCR signal transduction reveals new strategies for engaging established receptor targets to bring about distinct signaling and physiological outcomes. Additionally, this research is continuing to identify new GPCR regulatory proteins with both therapeutic and prognostic potential. Understanding the diversity of possible GPCR signaling outcomes, driven via ligand bias and/or system bias, will facilitate the development of pathway-selective GPCR-targeted therapeutics. The ability to monitor and quantify GPCR signaling via multiple pathways and across levels of evaluation, from regionally restricted second messengers/effectors to cellular, physiological, and behavioral responses, will be critical to this effort. We thank the authors for contributing their outstanding work to this Research Topic.

